# Risk factors influencing contamination of customized cosmetics made on-the-spot: Evidence from the national pilot project for public health

**DOI:** 10.1038/s41598-020-57978-9

**Published:** 2020-01-31

**Authors:** Hye Won Kim, Yae Sle Seok, Tae Jin Cho, Min Suk Rhee

**Affiliations:** Department of Biotechnology, College of Life Sciences and Biotechnology, Korea University, Seoul, 02841 Republic of Korea

**Keywords:** Environmental microbiology, Health care

## Abstract

Customized cosmetics made by consumers or sellers on-the-spot have several safety issues, and therefore require a preventative approach to their safety management. The present study aimed to identify potential factors affecting the safety of customized cosmetics made on-the-spot. Heavy metals and microbial contaminants in customized cosmetics were analyzed in 120 samples. It was revealed that the transfer of cosmetics to new containers during the production process is a significant risk factor for cross-contamination and that heat treatment is crucial for reducing the number of microorganisms in the products. For instance, cosmetics made with heat and with no transfer showed relatively low microbial counts ranging from not detected to 440 CFU/ml. The high pH (>pH 10) of samples did not guarantee the microbial safety of the freshly made cosmetics (with a rinse-off product having 2,830 CFU/ml and a pH of 11.2). There was no significant difference in microbial counts among cosmetic types (*P* > 0.05); however, semisolid types, especially creams and rinse-off products, were susceptible to contamination (maximum 2,710 and 2,830 CFU/ml, respectively). Most microorganisms in the customized cosmetics (40.8%) decreased to non-detectable levels during 60 days of storage. None of the samples harbored heavy metals. Sequencing analysis of isolates revealed some bacteria and mold that could cause human infections. The results of this study suggest that the regulation of customized cosmetics should consider the risk factors revealed in this study, as the products made on-the-spot are also final products sent directly to consumers.

## Introduction

South Korea, the 8^th^ largest cosmetic market in the world^1^, is at the forefront of the customized cosmetic trends. In 2016, the South Korean government conducted a pilot project on customized cosmetics targeting products made on-the-spot by readily producing bases and ingredients for colors, scents, and functionalities according to the consumer’s preference^2^. Customized cosmetics, such as consumer-fit skincare products, makeup products, and perfume, have already been sold in Europe, the USA, Canada, Australia, etc^3–6^. This pilot project was the world’s first governmental activity for the assessment and development of customized cosmetics made on-the-spot by consumers or sellers in the market.

Cosmetics are important vehicles of transmission of pathogens in people’s daily lives^7^. Most cosmetics, including customized cosmetics, contain an abundant amount of water and nutrients^8^, thereby providing good substrates for the survival of a variety of microorganisms^9^. The microbial contamination of cosmetics and the survival of microorganisms deteriorate the quality of the cosmetic and seriously affect human health^8,10,11^. Although safety issues resulting from contaminated cosmetics are currently rare^12^, product recalls still occur worldwide due to microbial contamination of the products^13,14^.

Cosmetics are not required to be sterile; however, the safety of the products after they have been sold to consumers also has to be assured^15^. To combat bacterial contamination and growth in cosmetics, the microbiological stability and durability of commercial cosmetics are tested before launch. However, the preservation system of customized cosmetics made-on-the spot cannot be assured as they are made on site and sent directly to consumers. The production process of on-the-spot cosmetics is also complicated (Fig. S1) and includes potential risks for cross-contamination from the surrounding environment, utensils, or handlers. Accordingly, these cosmetics can be susceptible to microbial contamination during the production process. Thus, microbial contamination of these novel cosmetics and their regulation is a concern for the public, manufacturers, and the government.

As customized cosmetics made on-the-spot are regarded as general commercial cosmetics, final products should comply with microbial quality and heavy metal limits. Although there are currently no internationally harmonized limits, it is generally accepted that total aerobic microbe counts of cosmetics for children under the age of three should be below 100 CFU/g or ml; those of the other cosmetic products should be below 1,000 CFU/g or ml in Europe, the USA, and South Korea^16,17^. The absence of *E. coli, P. aeruginosa*, and *S. aureus*, which are described as specified microorganisms that are generally considered undesirable in cosmetic products, is required in cosmetics^18^. Heavy metals (lead, arsenic, mercury, antimony, and cadmium) should also be managed according to the regulations^18^.

In the present study, to provide a comprehensive understanding of the potential microbial and chemical risks of customized cosmetics made on-the-spot, all shops participating in the national pilot project on customized cosmetics available in South Korea were investigated. Multifaceted approaches were applied to identify potential factors affecting the safety of customized cosmetics made on-the-spot, including preparation methods, pH, cosmetic types, forms, and storage (stability test) of the products. Heavy metals and microbial prevalence in the total of 120 customized cosmetic samples were also analyzed to evaluate the current safety of the products.

## Results and Discussion

The US FDA states that cosmetics need not be sterile; however, cosmetic products must not be contaminated with pathogenic microorganisms, and the level of nonpathogenic microorganisms should be low^12^. Achieving this standard requires a good quality management system for cosmetics, which consists of raw material quality and hygienic design of facilities^19^. Accordingly, good manufacturing practice (GMP) has been implemented over the decades to improve the industrial quality control of cosmetics^11^. However, GMP regulations are not appropriate for customized cosmetics made on-the-spot because this type of cosmetics can be made by untrained customers or sellers in shops with no specialized facilities for the manufacture of cosmetics. Customized cosmetics, therefore, are at risk of microbial contamination during the production process. The current study targeted all customized cosmetics made on-the-spot by the national pilot project in South Korea. Because the number of customized cosmetic products can be innumerable according to the mixed ingredients, the plan for sample collection was designed with the following criteria based on a previous field survey: i) to include a formula that is recommended by seller, ii) to include well-known customized cosmetic formulas (the most frequently sold products), iii) to include all raw materials used in the shops, and iv) to include the maximum number of raw materials that can be combined in the final products. Finally, a total of 120 customized cosmetic samples consisting of nine types, and three forms were collected directly from shops and analyzed.

**Figure 1:**
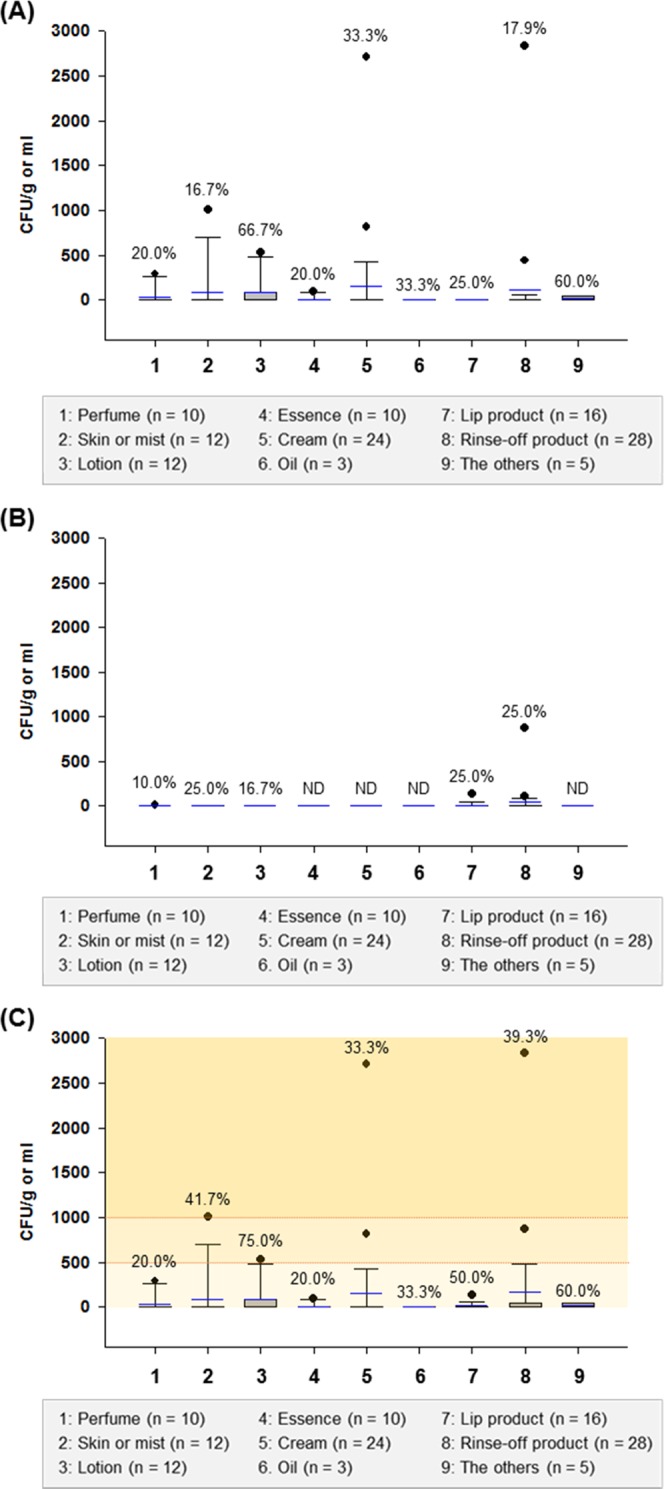
Box plots of (**A**) total bacterial counts, (**B**) total mold and yeast counts, and (**C**) total aerobic microbe counts of nine types of customized cosmetics (n = 120): perfume (n = 10), skin or mist (n = 12), lotion (n = 12), essence (n = 10), cream (n = 24), oil (n = 3), lip product (n = 16), rinse-off product (n = 28), and the others (n = 5). Square means of the interquartile range for each data point, and the black and blue lines indicate the median and mean values, respectively. The error bars above and below the square denote the 90^th^ and 10^th^ percentiles, and the black circles represent outliers. Values (%) on the bars indicate the detection rate of each microorganism in the samples, and ND indicates no detection of microorganisms.

The microbiological prevalence of freshly made cosmetic samples was clear (Fig. 1). Aerobic microbes were detected in a total of 49 samples (40.8%), and the microbial counts of the positive samples ranged from 10–2,830 CFU/g or ml. According to the microbial limit specified in the national regulations^18^, the total aerobic microbe counts of cosmetics for children under the age of three should be below 100 CFU/g or ml; those of other cosmetic products should be below 1,000 CFU/g or ml. This limit is considered high enough to prevent rejection of most contaminated cosmetic products. In this study, three customized cosmetics (a rinse-off product, baby lotion, and moisturizer) did not comply with the standard limit (2,830 CFU/ml, 530 CFU/ml, and 2,710 CFU/ml, respectively) (Table S2).

**Figure 2:**
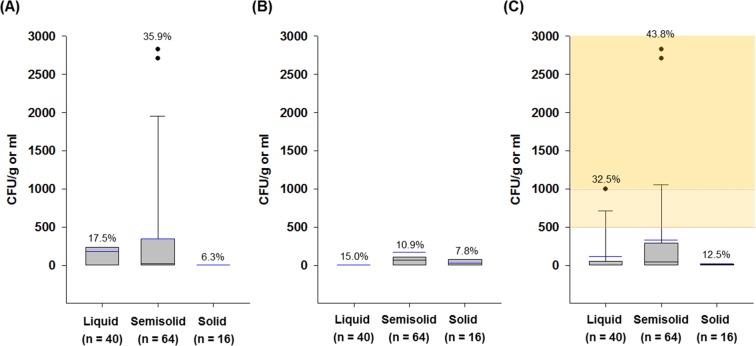
Box plots of (**A**) total bacterial counts, (**B**) total mold and yeast counts, and (**C**) total aerobic microbe counts of different formulations of customized cosmetics (n = 120): liquid (n = 40), semisolid (n = 64), and solid (n = 16). Square means of the interquartile range for each data point, and the black and blue lines indicate the median and mean values, respectively. The error bars above and below the square denote the 90^th^ and 10^th^ percentiles, and the black circles represent outliers. Values (%) on the bars indicate the detection rate of each microorganism in the samples, and ND indicates no detection of microorganisms.

The results of this study are similar to those of many other studies on commercial cosmetics, which reported microbial counts in creams, lotions, bath foam, and shampoo ranging from 10^2^ to 10^4^ CFU/g or ml^8,11,13,20,21^. Although there was no significant difference in the microbial counts of customized cosmetics according to cosmetic types (*P* > 0.05) (Figs. 1 and 2), creams that are generally recognized as being susceptible to microbial contamination also showed high aerobic microbe counts (Fig. S2), and therefore require proper quality control during preparation at the shop.

**Figure 3:**
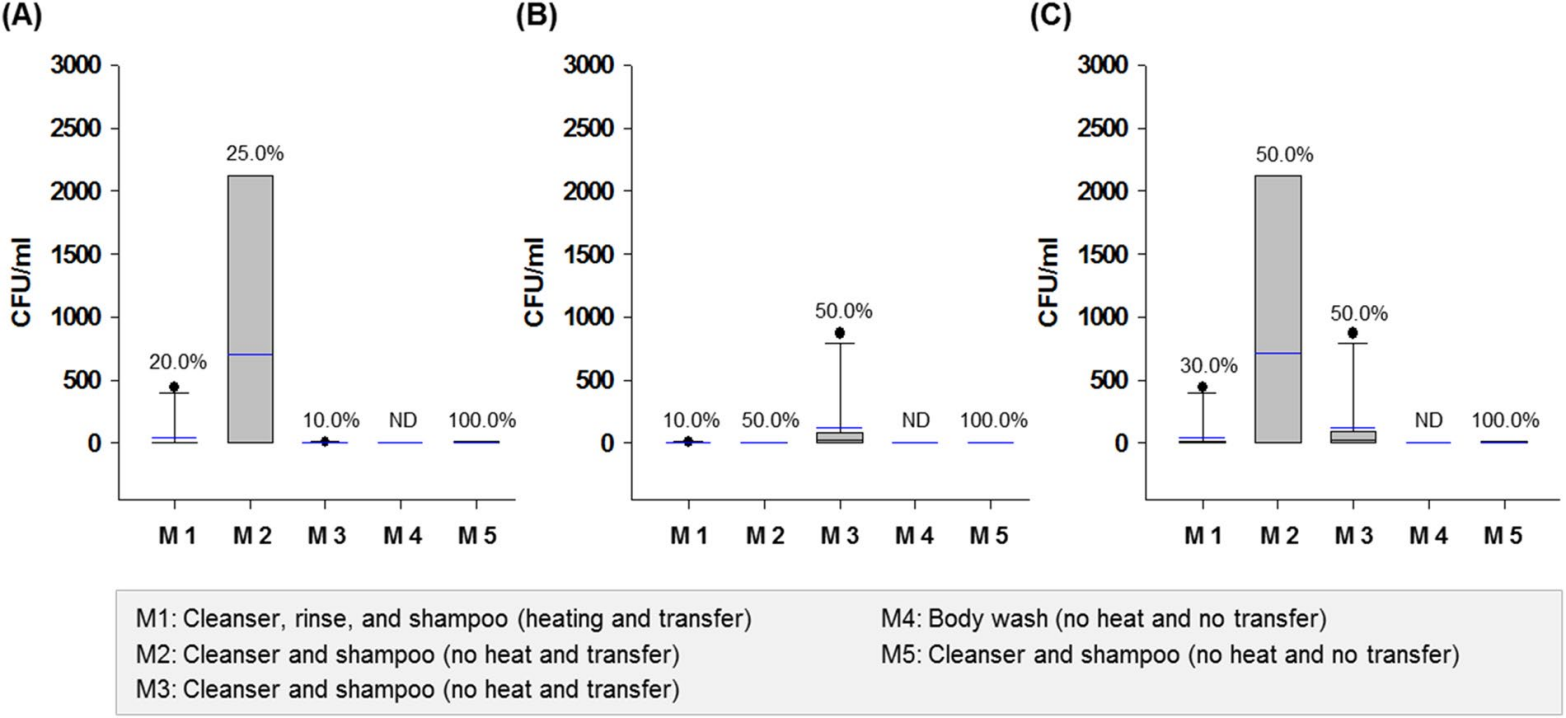
Box plots of (**A**) total bacterial counts, (**B**) total mold and yeast counts, and (**C**) total aerobic microbe counts of rinse-off products according to the preparation methods, M1 to M5. Square means of the interquartile range for each data point, and the black and blue lines indicate the median and mean values, respectively. The error bars above and below the square denote the 90^th^ and 10^th^ percentiles, and the black circles represent outliers. Values (%) on the bars indicate the detection rate of each microorganism in the samples, and ND indicates no detection of microorganisms.

The most specific characteristic of customized cosmetics is the preparation process at the shop. Unlike cosmetic manufacturing plants, customized cosmetic shops use small-scale equipment for heating and mixing ingredients or manual mixing of the ingredients with rods or by shaking; mixed products are sometimes transferred to new cosmetic containers using a spatula or not (Fig. S1). In this study, customized cosmetics (rinse-off products) made with heating (M1) and without transfer (M4 and M5) showed relatively low microbial counts (ND to 440 CFU/ml) (Fig. 3). However, cosmetics made with no heating and that were transferred to new containers (M2 and M3) showed increased microbial counts (ND to 2,830 CFU/ml). These results clearly indicate that heating is a crucial factor for reducing microorganisms in customized cosmetics and that the transfer of cosmetics to new containers could result in cross-contamination.

**Figure 4:**
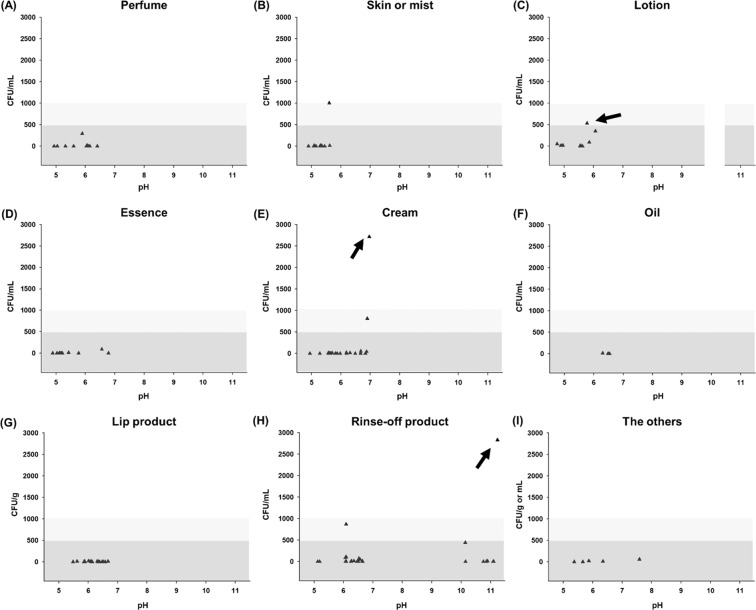
Dot plots of microbial populations of nine types of customized cosmetics (n = 120) according to pH: (**A**) perfume, (**B**) skin or mist, (**C**) lotion, (**D**) essence, (**E**) cream, (**F**) oil, (**G**) lip product, (H) rinse-off product, and (**I**) the others. The arrow indicates cosmetic samples that did not comply with the standard microbial limits.

It was also well known that cosmetic products with pH values below 3 or above 10 are considered to have a low risk of microbial growth^22^; however, it was shown that customized cosmetics with pH values above 10 contained microorganisms in this study (Figs. 4 and S3). As customized cosmetics are made on-the-spot, the high pH condition may have not greatly affected the survival of the contaminated microorganisms. However, the freshly made cosmetics are also final products; thus, the improved hygienic control of customized cosmetics, even high-pH cosmetics, should be considered.

**Figure 5:**
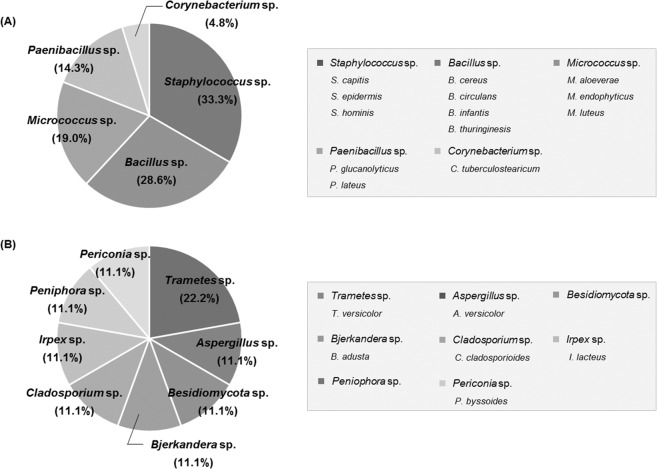
Pie chart representation of the genera of (**A**) bacteria and (**B**) mold and yeast isolated from the customized cosmetics. Microorganisms presented in the gray box indicate the isolated species of each genus.

Several studies have shown that the most frequently found microorganisms in cosmetics are *P. aeruginosa, S. aureus, E. coli*, and *Bacillus* species, as well as other bacteria, mold and yeasts^8–10,23,24^. In this study, we used traditional testing methods for specified microorganisms by using enrichment and selective medium; however, no target bacteria were isolated. Instead, we isolated and identified certain microorganisms, which cannot be isolated by the traditional testing methods, in customized cosmetics by 16S rRNA and ITS sequencing (Fig. 5). Most isolates of the study were identified as safe microorganisms that are observed on normal skin or are ubiquitous in nature, such as soil, water, and air. However, some bacteria and molds that cause human infections were also observed, such as *S. epidermidis*, *B. cereus*, *B. circulans*, and *A. versicolor*.

Some species of *Staphylococcus*, such as *S. epidermidis*, could cause skin infections such as desquamate and acne^25^. *Bacillus*, the second most prominent genus, are transient skin microflora^26^, and *B. cereus* contamination in eye cosmetics could also cause severe eye infections^25^. Fungal multiplication in cosmetics is more rapid and evident than bacteria, and fungal infections can be caused by *Candida, Aspergillus*, and *Penicillium* species in particular^7,23^. Most isolated fungi in this study were not pathogenic, generally being those found in food and the environment, and used as cosmetic ingredients in some cases (e.g., *Trametes versicolor*); however, some are not good for human health. For instance, *A. versicolor* is an eye, nose, and throat irritant as are other *Aspergillus* species.

The microbial contaminants of customized cosmetics can originate from i) contaminated raw materials, ii) the specific production process of customized cosmetics, iii) the sanitary conditions of the environment and equipment (i.e., improper air conditioning of the shop, reuse of utensils, etc.) and iv) poor personal hygiene. Though microbial populations in some raw materials, air, hand, and utensil samples were quite low in the limited study (Tables S3 and S4), phylogenetic tree showed that same bacterial species were isolated from different sources (Fig. S4). For example, *Bacillus* species were observed in raw materials and final products. Therefore, the risk factors mentioned above should be monitored continuously and be managed by a regulation for the preparation and sale of customized on-the-spot cosmetics.

**Figure 6:**
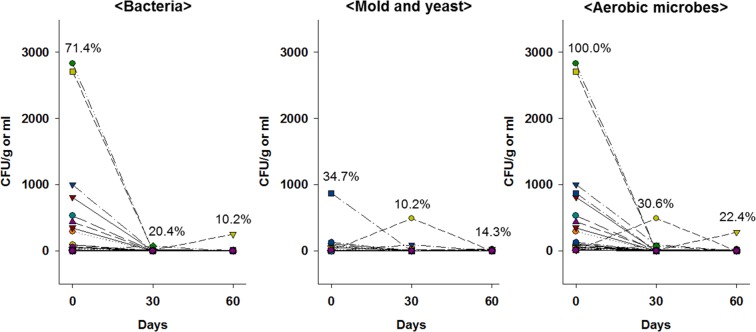
Survival of bacteria, mold and yeast, and aerobic microbes in microbiologically positive customized cosmetic samples (n = 49) after storage up to 60 days at 22 °C. Values (%) on the graph indicate the detection rate of each microorganism in the samples.

It is also important to identify the microbiological stability and durability of these contaminated customized cosmetics through the storage period to determine their potential risk during consumer use. Here, the survival of bacteria and yeast and mold in 49 customized cosmetics was tested for up to 60 days (Fig. 6). Because most aerobic microbes decreased to the ND level or the residual microbial counts were reduced, the preservative system of customized cosmetics is considered safe. However, as the preservative effect of customized cosmetics cannot be verified before consumer use, sellers could test possible combinations of customized cosmetics in advance, could display the information of customized cosmetics (i.e., ingredients used, lot number of raw materials, mixing ratios, etc.), or could store and test the remains of cosmetic samples sold.

According to our previous consumer survey, one of the major safety issues of customized cosmetics is chemical reactions when mixing the ingredients at the shops^5^. However, experts in an advisory committee for cosmetics pointed out heavy metals in the final cosmetic samples are more important than chemical reactions because most ingredients used for customized cosmetics are safe materials that can be mixed with various other ingredients. In this study, five types of heavy metals, lead, arsenic, mercury, antimony, and cadmium, were tested in 120 customized cosmetic samples, and none of the samples harbored heavy metals.

## Conclusions

In conclusion, while the sanitary conditions of the environment, utensils, and handlers of the customized cosmetic shops were determined to be of good status by a preliminary survey, high microbial counts and the presence of microorganisms in the final products still indicates possible microbial contamination during the production of customized cosmetics. The results of this study clearly identified risk factors for cosmetic contamination; semisolid type products, including cream and rinse-off products, are the most susceptible to microbial contamination; pH values of cosmetics greater than 10 did not guarantee low microbial counts in the case of freshly made customized cosmetics; the major risk factors for microbial contamination of customized cosmetics made on-the-spot are thought to be raw materials and the transfer of cosmetics to new containers; and heat treatment during the process is a critical control point that could affect the microbiological levels of customized cosmetics. The empirical results provided herein may help authorities establish legislation and regulations for customized cosmetics and help cosmetic manufacturers and sellers to design strategic management plans to improve the quality and safety of customized on-the-spot cosmetics. Indeed, South Korea government announced that regulations for the customized cosmetics made on-the-spot would be enforced in 2020.

## Methods

### Sample collection

A total of 120 commercially available customized cosmetics, processed in the shops by consumers or sellers just before sale, were collected from March to September 2016 in Seoul, South Korea. The composition of nine different cosmetic types and the number of customized cosmetics are presented in Table S1. Briefly, perfume (n = 10), skin or mist (n = 12), lotion (n = 12, including lotion for babies), essence (n = 10), cream (n = 24), oil (n = 3), lip products (n = 16), rinse-off products (n = 28; cleansing oil, foam cleanser, hand cleanser, shampoo, conditioner, and body cleanser) and other products (n = 5; clay mask, mask pack, and peels and scrubs) were collected and subjected to microbiological and chemical analyses immediately after delivery to the laboratory (within 1 hour).

### Microbiological testing of customized cosmetics

All microbiological analyses were performed according to the US Food and Drug Administration Bacteriological Analytical Manual: Microbiological Methods for Cosmetics^17^ and the Koreas regulations on safety standards, etc. of cosmetics^18^, with some modifications.

#### Microbial counts: bacteria and yeast/mold

For water-miscible products, 1 ml or g of the well-mixed sample was transferred to a dilution tube containing 9 ml of modified Letheen broth (MLB, Difco, Sparks, MD, USA). For water-immiscible products, 1 ml or g of the sample was transferred to a dilution tube containing 8 ml of MLB and 1 ml of polysorbate 80 and then homogenized. If the sample was not well dispersed, glass beads were added and placed in a water bath at 40 °C for 20 min and vortexed. Homogenized samples were serially diluted using 10-fold dilution methods with 9 ml of 0.85% sterile saline. One hundred microliters of diluent was spread-plated on modified Letheen agar (MLA, Difco) and potato dextrose agar (PDA, Difco) in duplicate. To reduce the detection limit to 10 CFU/ml or g, 1 ml of the sample was spread plated onto three plates. MLA and PDA were incubated at 35 °C for 24 hours and at 20 °C for 5 days, respectively. The number of colonies on the plates was then counted and calculated as CFU/ml or g. The number of aerobic microorganisms was calculated based on the total number of bacteria and fungi.

### *Presence of specified microorganisms:* Escherichia coli, Pseudomonas aeruginosa*, and* Staphylococcus aureus

To examine the specified microorganisms in the customized cosmetics, 1 ml or g of the well-mixed sample was transferred to a dilution tube containing 9 ml of lactose broth (Difco) for *E. coli* or tryptic soy broth (TSB, Difco) for *P. aeruginosa* and *S. aureus*. For water-immiscible products, 8 ml of broth and 1 ml of Polysorbate 80 were used as described above. The mixture was homogenized and incubated at 37 °C for 24 hours. Next, one loopful of enriched culture was streaked onto selective agar plates and incubated for presumptive identification of specified microorganisms as follows: *E. coli* (eosin methylene blue agar, Difco), *P. aeruginosa* (cetrimide agar, Difco), and *S. aureus* (Baird-Parker agar, Difco) at 37 °C for 24 hours. Typical colonies were isolated and confirmed by VITEK2 (bioMerieux) with GN (*E. coli*), ANC (*P. aeruginosa*), and GP (*S. aureus*) cards.

### Genomic DNA isolation, sequencing, and phylogenetic analysis

Isolates from the quantitative microbiological analysis of customized cosmetics were Gram stained to confirm the bacterial type. Briefly, a smear made from an emulsified colony was air dried and heat fixed. It was covered with crystal violet for 60 sec and washed off. The smear was then covered with lugol’s iodine for 60 sec, washed off, and decolorized with acetone alcohol for 15 sec. It was washed off again and counter stained with safranin for 60 sec. After washing, the smear was examined using microscopy (Leica DM6000, Leica Microsystems GmbH, Mannheim, Germany). Genomic DNA was then extracted using a LaboPass mini kit (Cosmogenetech, Seoul, South Korea) according to the manufacturer’s instructions. The 16S rRNA gene of bacteria and the internal transcribed spacer (ITS) region of fungi was amplified by PCR with the universal primers 27 F (5′-AGAGTTTGATCCTGGCTCAG-3′) and 1492 R (5′-GGTTACCTTGTTACGACTT-3′) for bacteria^27^ and ITS1 (5′-TCCGTAGGTGAACCTGCGG) and ITS4 (5′-TCCTCCGCTTAT TGATATGC) for fungi^28^. Cloned PCR amplicons were purified using a MEGAquick-spin Total Fragment DNA Purification kit (iNtRON Biotechnology, South Korea) and then sequenced by the Cosmogenetech Sequencing Service (Cosmogenetech). Briefly, sequencing reactions were performed using BigDye terminator PCR with the following conditions: predenaturation at 94 °C for 1 min, 25 cycles of denaturation at 94 °C for 20 sec, annealing at 50 °C for 15 sec, and extension at 60 °C for 4 min. The gene sequence was then analyzed with BLAST sequence analysis software (https://blast.ncbi.nlm.nih.gov/Blast.cgi) using the National Center for Biotechnology Information (NCBI) server.

### pH measurement

Customized cosmetic products for pH measurement were prepared by adding 2 ml or g of the sample to 30 ml of distilled water, that is to say we measured the pH of the diluted sample. The diluents were heated in a water bath at 40 °C to dissolve and mix the fat. The pH of the samples was measured using an S20 SevenEasy pH meter (Mettler Toledo, Schwerzenbach, Switzerland) at room temperature. The pH meter was calibrated sequentially before measurement with commercially available three-point standard buffer solutions at pH 4.01, 7.00, and 10.01 (Mettler Toledo).

### Determination of lead, arsenic, mercury, antimony, and cadmium

Heavy metals in customized cosmetic products were analyzed by an authorized laboratory for cosmetic inspection in South Korea (Korea Advanced Food Research Institute). Briefly, lead (Pb) and arsenic (As) were determined using inductively coupled plasma – optical emission spectrometry (ICP-OES Optima 8300DV, Perkin Elmer, USA), mercury (Hg) was measured by a direct thermal decomposition mercury analyzer (NIC MA-3000, Nippon Instrument Corporation, Japan), and antimony (Sb) and cadmium (Cd) were analyzed by ICP-MS (icapQ, Thermo Science, USA).

### Statistical analysis

Microbial counts of samples tested in this study were statistically analyzed using SAS software version 9.4 (Statistical Analysis Systems Institute, Cary, NC, USA; https://www.sas.com/en_us/software/sas9.html). Data were examined using a general linear model for analysis of variance, and Tukey’s test was used to determine the significance of differences (*P* < 0.05).

## Supplementary information

Supporting Information.
