# Author Correction: Risk factors influencing contamination of customized cosmetics made on-the-spot: Evidence from the national pilot project for public health

**DOI:** 10.1038/s41598-020-63867-y

**Published:** 2020-04-30

**Authors:** Hye Won Kim, Yae Sle Seok, Tae Jin Cho, Min Suk Rhee

**Affiliations:** Department of Biotechnology, College of Life Sciences and Biotechnology, Korea University, Seoul, 02841 Republic of Korea

Correction to: *Scientific Reports* 10.1038/s41598-020-57978-9, published online 31 January 2020

This Article contains typographical errors in the Acknowledgements section.

“This research was supported by a grant from the Korean Ministry of Food and Drug Safety (15162MFDS053)”

should read:

“This research was supported by a grant from the Korean Ministry of Food and Drug Safety (16172MFDS240)”

