# Supplementary material for: Risk factors influencing contamination of customized cosmetics made on-the-spot: Evidence from the national pilot project for public health
Source: Sci Rep. 2020 Jan 31;10:1561. doi: 10.1038/s41598-020-57978-9 (PMC6994525; doi:10.1038/s41598-020-57978-9)
Supplement: Supplementary file 1 — Supporting Information. [file 41598_2020_57978_MOESM1_ESM.docx]

**[Supplementary materials]**

**Supplementary method**

**S1. Raw materials, air, hand, and utensils.** Microorganisms in raw materials (with the same lot number for the final products), air, producers’ hands, and utensils including container and spatula were analyzed in case the customized cosmetics exceeded the standard limit of microorganisms. Microbial counts in raw materials were tested with the same method for cosmetics as described above. Workplace air was tested by air sampler (Aquaria Microflow, Milan, Italy) according to the manufacturer’s instruction. Air samples were collected by operating machine for 5 min (100 L/min absorption). Collected samples in medium were then incubated under the appropriate condition. Colonies were counted and the unit was converted to CFU/L. Meanwhile, producers’ hands, containers for cosmetic processing, and spatulas were analyzed by swab method. Briefly, a 5 × 5 cm^2^ frame was placed on the surface of the sample and the surface was rubbed with a cotton swab. A cotton swab was put into 5 ml sterile saline and vortexed for 1 min to make a sample solution. And then 1 ml of the sample was placed in 9 ml of sterile saline and 10-fold diluted. Total 1 ml of the sample solution and 100 μl of the diluted solution were plated in a suitable medium and cultured. Colonies were counted and the unit was converted to CFU/cm^2^. In the case of the analysis of producers’ hands, study protocols were approved by the ethical committee of Institute of Biomedical Science and Food Safety in Korea university and written informed consent were obtained by all participants. Experiments were conducted in accordance with relevant guidelines and regulations.

**S2. Phylogenetic analysis**

A phylogenetic tree was constructed using the neighbor-joining method as well as maximum-likelihood from evolutionary distances by using MEGA software version 7.0.26 (MEGA, PA, USA). The topology of the tree was assessed by performing bootstrap analysis with 1,000 replications.

**Table S1.** Composition of nine different types of customized cosmetic products purchased from five shops

| **Samples** | **No. of samples tested** | | | | | |
| --- | --- | --- | --- | --- | --- | --- |
|  | **Shop A** | **Shop B** | **Shop C** | **Shop D** | **Shop E** | **Total** |
| 1. Perfume | 0 | 0 | 10 | 0 | 0 | 10 |
| 2. Skin or mist | 6 | 0 | 0 | 6 | 0 | 12 |
| 3. Lotion | 6 | 0 | 0 | 6 | 0 | 12 |
| 4. Essence | 5 | 0 | 0 | 5 | 0 | 10 |
| 5. Cream | 12 | 0 | 0 | 12 | 0 | 24 |
| 6. Oil | 3 | 0 | 0 | 0 | 0 | 3 |
| 7. Lip product | 1 | 15 | 0 | 0 | 0 | 16 |
| 8. Rinse-off product | 7 | 0 | 0 | 7 | 14 | 28 |
| 9. The others | 3 | 0 | 0 | 2 | 0 | 5 |
| **Total** | **43** | **15** | **10** | **38** | **14** | **120** |

**Table S2.** Customized cosmetic samples above the standard microbial limit

| **No.** | **Cosmetic type** | **Shop** | **pH** | **The corresponding**  **standard microbial limit ^a^** | **Microbial population (CFU/ml)** | | | **Preparation method** |
| --- | --- | --- | --- | --- | --- | --- | --- | --- |
|  |  |  |  |  | **Bacteria** | **Mold and yeast** | **Aerobic microbes** ^b^ |  |
| 1 | Rinse-off product  (body cleanser) | A | 11.23 | <1,000 CFU/g(ml) | 2,830 | ND ^c^ | 2,830 | M 2 |
| 2 | Lotion  (for babies) | A | 5.78 | <100 CFU/g(ml) | 530 | ND | 530 | M 1 |
| 3 | Cream  (moisturizing cream) | D | 6.96 | <1,000 CFU/g(ml) | 2,710 | ND | 2,710 | M 1 |

^a^, Standard microbial limit based on International Organization for Standardization (ISO) and Korean regulations.

^b,^ Bacteria, mold and yeast.

^c^,ND, not detected (detection limit = 10 CFU/g or ml).

Table S3. Microbial populations in raw materials of samples above the standard microbial limit

| **Raw materials** | **Microbial population (CFU/ml)** | | |
| --- | --- | --- | --- |
|  | **Bacteria** | **Mold and Yeast** | **Aerobic microbe** ^a^ |
| **Shop A:** | | | |
| **Rinse-off product (body cleanser)** |  |  |  |
| Cleanser base | ND | ND | ND |
| Ingredient (average) |  |  |  |
| Pomegranate extract | ND | ND | ND |
| Spirulina extract | 70 | ND | 70 |
| Acerola extract | ND | 10 | 10 |
| Geranium water | 10 | ND | 10 |
| Chamomile water | ND | ND | ND |
| **Lotion (for babies)** |  |  |  |
| Lotion base | ND | ND | ND |
| Ingredient (average) |  |  |  |
| Aloe vera extract | 10 | ND | 10 |
| Horsetail extract | 10 | ND | 10 |
| Tea tree water | ND | ND | ND |
| Nolly water | 10 | ND | 10 |
| Grapefruit water | 30 | ND | 30 |
| Avocado oil | ND | ND | ND |
| Olive oil | ND | ND | ND |
| **Shop D:** | | | |
| **Cream** |  |  |  |
| Cream base | ND ^b^ | ND | ND |
| Ingredient (average) |  |  |  |
| Mung bean extract | 10 | ND | 10 |
| Lotus extract | ND | ND | ND |
| Centipede extract | ND | 20 | 20 |
| Bamboo leaf extract | 10 | ND | 10 |
| Arkhorn | ND | ND | ND |
| Kappa peptide | ND | ND | ND |

^a^ Aerobic microbe, Bacteria, mold and yeast.

^b^ ND, not detected (Detection limit = 10 CFU/g or ml).

**Table S4.** Microbial populations in air, hand, utensils in shops selling product above the standard microbial limit

| **Sample** | **Microbial population** | | |
| --- | --- | --- | --- |
|  | **Bacteria** | **Mold and Yeast** | **Aerobic microbe** ^a^ |
| **Shop A:** | | | |
| Air | 0.02 CFU/L | ND | 0.02 CFU/L |
| Hand | ND | ND | ND |
| Container for processing | ND | ND | ND |
| Spatula | 0.2 CFU/cm^2^ | ND | 0.2 CFU/cm^2^ |
| **Shop D:** | | | |
| Air | 0.006 CFU/L | ND | 0.006 CFU/L |
| Hand | 0.2 CFU/cm^2^ | ND | 0.2 CFU/cm^2^ |
| Container for processing | ND | ND | ND |
| Spatula | ND | ND | ND |

^a^ Aerobic microbe, Bacteria, mold and yeast.

^b^ ND, not detected (Detection limit = 10 CFU/g or ml).

**
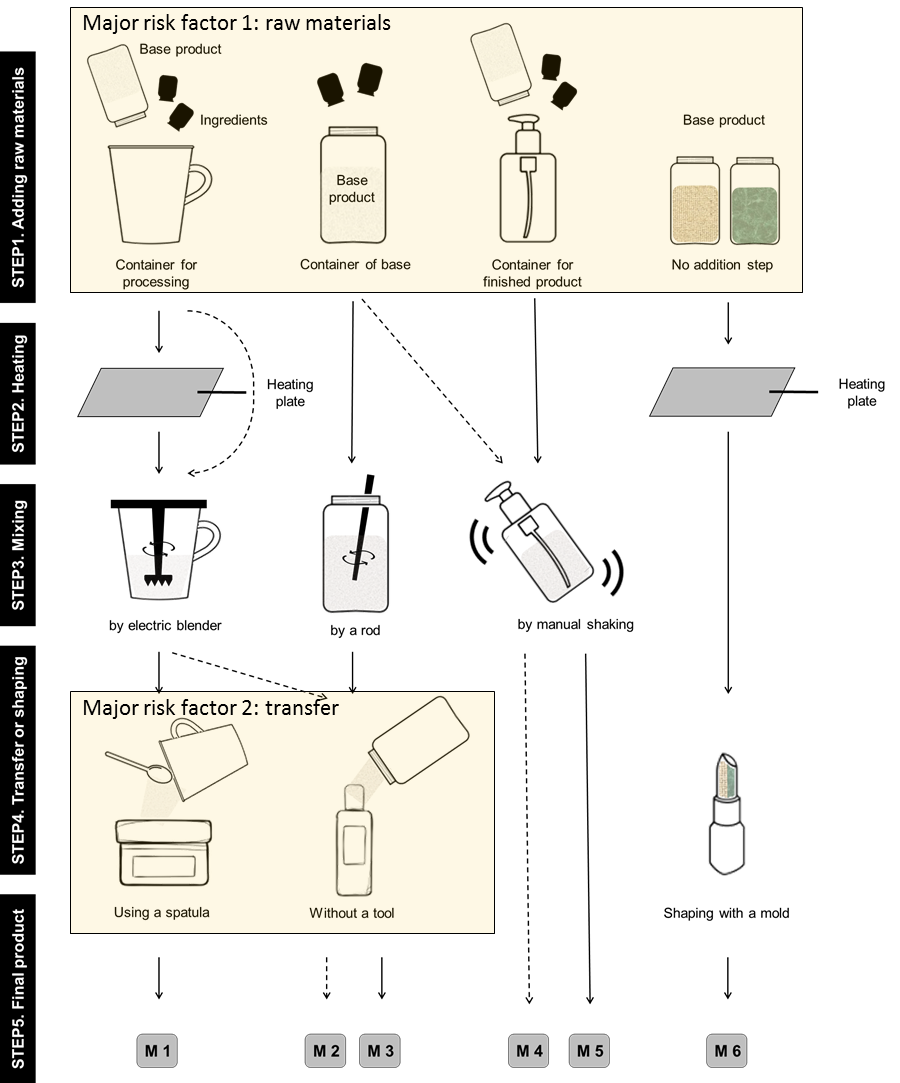
**

**Figure S1.** Five steps of the customized cosmetic production process: Step 1. Adding raw materials, Step 2. Heating, Step 3. Mixing, Step 4. Transfer or shaping, and Step 5. Final product. The methods for preparing customized cosmetics are classified into six categories, M1 to M6.

**
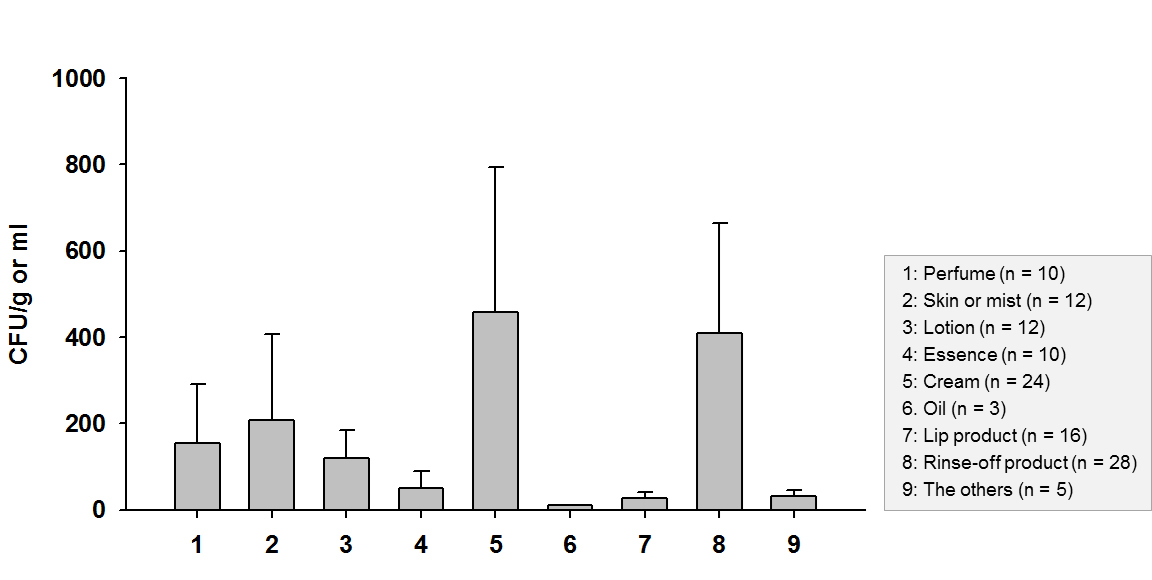
**

**Figure S2.** Total aerobic microbe counts of nine types of customized cosmetics (n = 120). Values indicate the mean ± standard error.

**
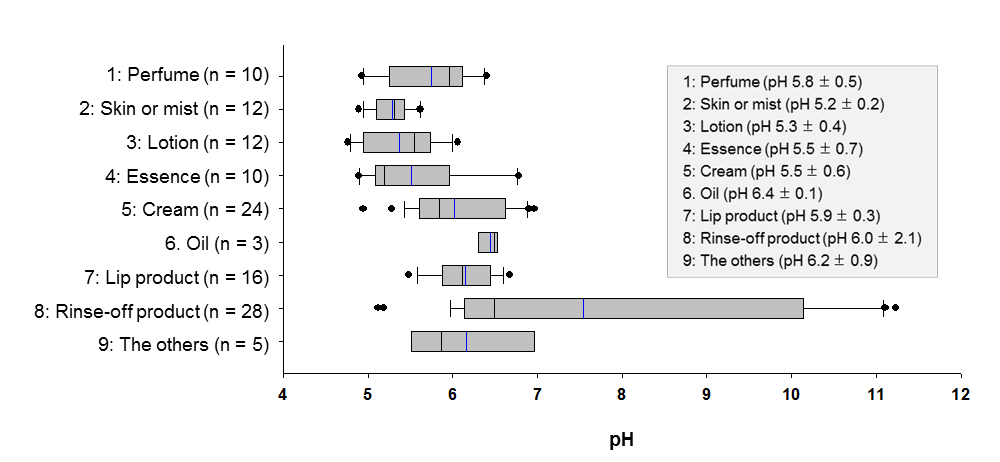
**

**Figure S3.** Box plots of pH of nine types of customized cosmetics: Square means of the interquartile range of each data point; the black and blue lines represent the median and mean values, respectively. The error bars above and below the square denote the 90^th^ and 10^th^ percentiles, and the black circles indicate outliers. pH values in the gray box indicate the mean ± standard deviation.

**
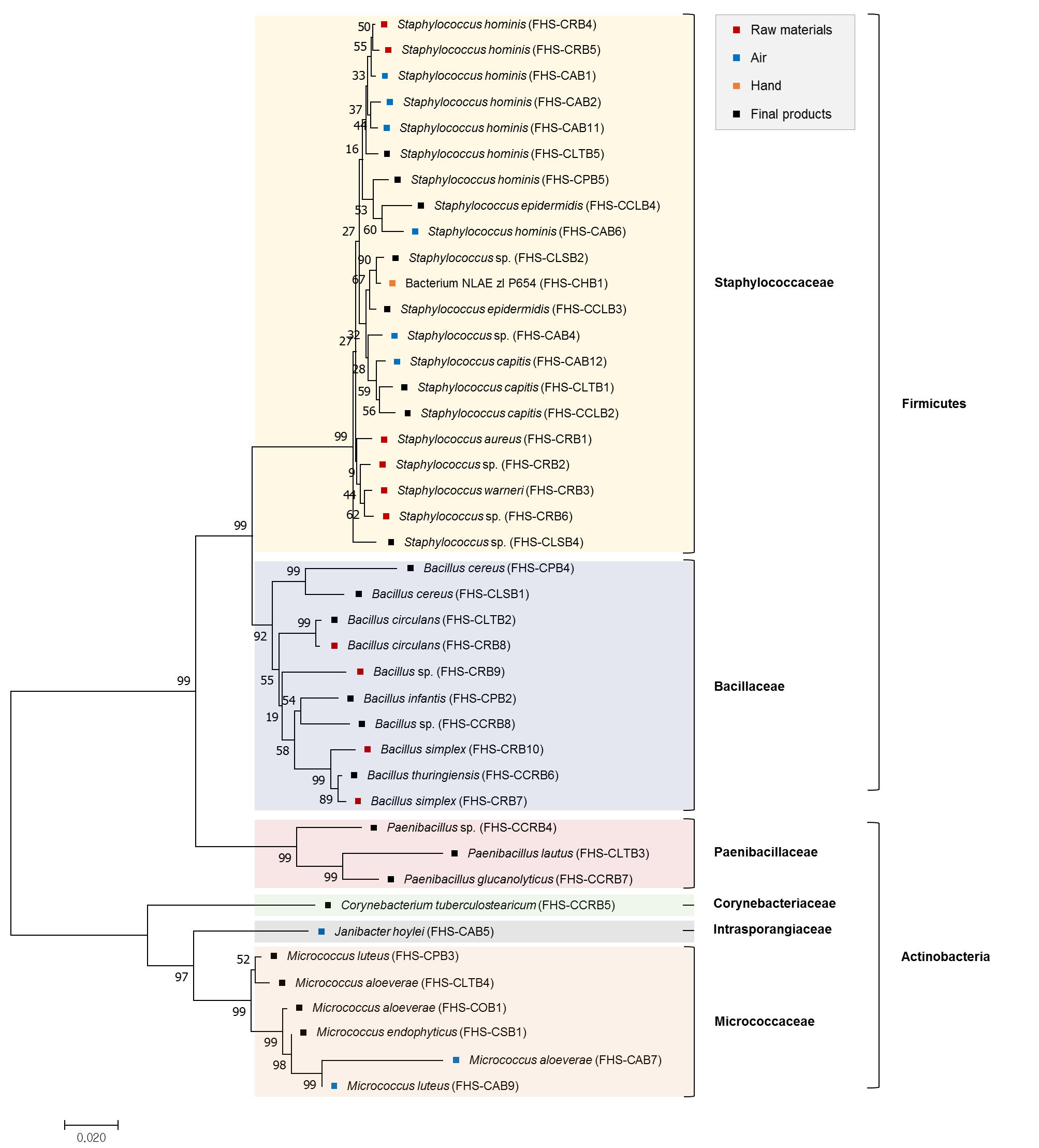
**

**Figure S4.** Phylogenetic tree showing the genetic relationships of the bacterial isolates from the raw materials (red), air (blue), hand (yellow), and final customized cosmetic products (black). The tree was constructed using the Neighbor-Joining method with the bootstrap test at 1,000 replicates by MEGA7. The scale bar represents 0.02 substitution per nucleotide position and the percentage of replicate trees are shown next to the branches. The colored shadow marks the different bacteria family.
